# Diagnostic competence and health worker knowledge of female genital schistosomiasis management in a rural Ghanaian district

**DOI:** 10.1371/journal.pntd.0013638

**Published:** 2026-06-30

**Authors:** Courage Gbeze, Akosua Bonsu Karikari, Samuel Amoako Asirifi, Nana Boakye Alahaman, Seth Christopher Yaw Appiah, Clémence Essé-Diby, Gloria Ivy Mensah, Kennedy Kwasi Addo

**Affiliations:** 1 Department of Clinical Microbiology, University for Development Studies, Tamale, Ghana; 2 Obstetrics & Gynaecology Department, Tamale Teaching Hospital, Tamale, Ghana; 3 Department of Sociology and Social Work, Kwame Nkrumah University of Science and Technology, Kumasi, Ghana; 4 Department of Research and Development, Centre Suisse de Recherches Scientifiques en Côte d’Ivoire, Abidjan, Côte d’Ivoire; 5 Department of Bacteriology, Noguchi Memorial Institute for Medical Research, University of Ghana, Accra, Ghana; University of Buea, CAMEROON

## Abstract

**Background:**

Schistosomiasis is a neglected tropical disease with a greater burden in Africa, including Ghana. Female Genital Schistosomiasis (FGS), a gynaecological manifestation of urogenital schistosomiasis, is often missed or misdiagnosed due to similarities with sexually transmitted infections and other gynaecological infections, with limited ease of health worker identification and diagnostic capability. This study assessed healthcare workers’ knowledge and diagnostic capacity for FGS in the Central Gonja District of Ghana.

**Methods:**

A quantitative cross-sectional study sampled 237 healthcare workers from 19 facilities near the Black and the White Volta rivers using a three-phase multistage sampling process. Data was collected via a self-administered Kobo Toolbox questionnaire, focusing on sociodemographic factors, whether facilities had functional screening tools for FGS and health workers’ capacity to diagnose and treat these conditions. Analysis was conducted in Statistical Package for Social Sciences (SPSS), employing descriptive statistics and Pearson’s chi-square tests to assess inferential associations between variables and health workers’ knowledge of FGS/schistosomiasis, which served as the main outcome variable.

**Results:**

The study involved health workers with a mean age of 31.6 ± 4.18 years, of whom 52.3% were male. Knowledge gaps were significant: only 30% (71/237) demonstrated good understanding of schistosomiasis and merely 16.9% (40/237) showed adequate knowledge of FGS. Despite 91.6% recognition of schistosomiasis (‘Bilharzia’), knowledge of genital manifestations lagged severely (FGS: 26.8%, MGS (Male genital schistosomiasis): 18.1%). While demographic factors showed no association, experienced staff demonstrated better FGS knowledge (p = 0.003). Critical health system deficiencies emerged; 74% of facilities lacked laboratories, 90% lacked praziquantel and 100% lacked FGS diagnostic capacity. Even among clinicians, < 43% knew standard FGS treatment and only 1/3 considered FGS in relevant diagnoses.

**Conclusion:**

In the Central Gonja District of the savannah region located in the northern part of Ghana, where schistosomiasis prevalence is classified as moderate (10–49%), healthcare workers demonstrated limited knowledge and diagnostic capacity regarding FGS. These findings highlight the need to integrate FGS into reproductive health guidelines, strengthen healthcare worker training and improve diagnostic resources for early detection and management in endemic settings.

## Author summary

Female Genital Schistosomiasis is a neglected yet serious health condition affecting women, especially in sub-Saharan Africa. It often goes undiagnosed or is mistaken for sexually transmitted or other gynaecological infections due to overlapping symptoms and limited awareness among healthcare providers. This study explored how well healthcare workers in the Central Gonja District of Ghana understand and diagnose FGS. Through a survey of 237 healthcare workers from 19 health facilities located near the Black and the White Volta rivers, we uncovered significant knowledge gaps; only a small number had a clear understanding of FGS and how to treat it. Most health facilities lacked the necessary resources, including diagnostic tools and essential medication like praziquantel. Our findings highlight an urgent need to train healthcare workers and integrate FGS into routine reproductive health services to improve detection and care for affected women.

## Introduction

Human schistosomiasis, or bilharzia, is a neglected tropical parasitic disease prevalent in communities lacking access to sufficient safe water and proper sanitation facilities [1]. It is among the WHO’s twenty-one neglected tropical diseases (NTDs), a recognised disease of poverty with significant public health challenge in low and middle-income countries [2]. Schistosomiasis has been reported in 78 countries worldwide, with almost 93% of the 207 million global cases being found in Africa [3–5]. The disease causes debilitating consequences that is second only to malaria, exerting a significant socioeconomic impact as one of the most detrimental parasitic diseases [6]. More than 779 million people are at risk of infection causing about 280,000 deaths annually and a global burden of 3.3 million disability-adjusted life years [5,7–9]

The eggs of the parasite elicit an inflammatory immune response, leading to acute or chronic illness. While the disease is rarely fatal, its chronic form can cause significant health issues. *Schistosoma mansoni* and *S. intercalatum* infections are commonly associated with abdominal pain, bloody diarrhoea, high fever, hepatomegaly and hepatic fibrosis [10]. Meanwhile, chronic infection with *S. haematobium* may result in haematuria, bladder irritation, bladder cancer, as well as painful and frequent urination [10]. Prolonged infection with *S. haematobium* may lead to Female Genital Schistosomiasis (FGS) which is a gynaecological disease characterised by the presence of schistosome eggs and/or distinctive pathology in the female reproductive system. Eggs trapped in urogenital tissues trigger an inflammatory response, resulting in various lesions within the genital tract of affected women and girls [11,12].

FGS is a significant public health concern yet remains an overlooked and silent epidemic [13]. Up to 56 million adolescent girls and women are estimated to be at risk of FGS [2,14] with majority resident in sub-Saharan Africa [15,16]. This NTD is not well studied and often not prioritised by local, regional and global health policymakers [17,18]. FGS is neither included in the training curricula of many healthcare professionals nor routinely considered in the syndromic management of vaginal discharge, infertility, or cervicovaginal lesions in most schistosomiasis-endemic regions of Africa. As a result, the condition is frequently overlooked or misdiagnosed at health centres, Community Health Planning and Services (CHPS) compounds, clinics and even higher-level facilities such as polyclinics and district hospitals [19,20]. Malawi is a notable exception, having recently integrated genital schistosomiasis into the management of Sexually Transmitted Infection (STI) [21]. In settings where such integration is lacking, FGS may be incorrectly diagnosed as an STI or even as cancer. Despite sustained control efforts including mass praziquantel administration and school health education by the Ghana Health Service, schistosomiasis endemicity persists in Ghana’s freshwater communities where water-sanitation infrastructure is inadequate [22,23]. The disease burden reflects systemic challenges in health worker capacity building, with insufficient post-curricular training on schistosomiasis pathology, especially FGS, resulting in underdiagnosis and mismanagement in endemic zones. This knowledge deficit contributes to low clinical awareness of FGS and its complications, even in endemic regions [23–25].

This misdiagnosis is further compounded when patients are treated for STIs, being administered other medications such as antibiotics or antifungals, which have minimal to no effect on FGS [24] instead of praziquantel, the drug of choice for treating all forms of schistosomiasis. In addition to physical complications, FGS could lead to outcomes such as miscarriage and infertility, which may significantly impact women’s mental health and social status [26]. In addition to these, misdiagnosis often results in repeated visits to health facilities, increasing the strain on both the patient and the healthcare system [24]. Although important FGS-related initiatives and research, including the FAST Package interventions [23], have been introduced in Ghana, existing studies [24,27] have largely been conducted in southern parts of the country. There remains a paucity of published data from northern Ghana, despite ongoing schistosomiasis transmission and distinct health system dynamics in this region. This geographical gap highlights the relevance of the present study in contributing context-specific evidence from northern Ghana to inform future FGS programming and scale-up efforts. This study sought to investigate the knowledge level and capacity of healthcare workers to diagnose and manage FGS in an endemic district of the Savannah region of Ghana.

## Method

### Ethical considerations

Ethical approval for this study was obtained from the Institutional Review Board (IRB) of the University for Development Studies (UDS), Tamale, Ghana on the 29^th^ of March 2024 (Approval Number: UDS/RB/030/24). Permission to conduct the study was granted by the District Director of Health Services and the heads of the participating health facilities. The study involved the collection of anonymous questionnaire data. Written informed consent was obtained from all participants before completing the questionnaire. Participant confidentiality and anonymity were maintained throughout the study, as no personal identifiers or sensitive personal data were collected.

### Study area

**Fig 1:**
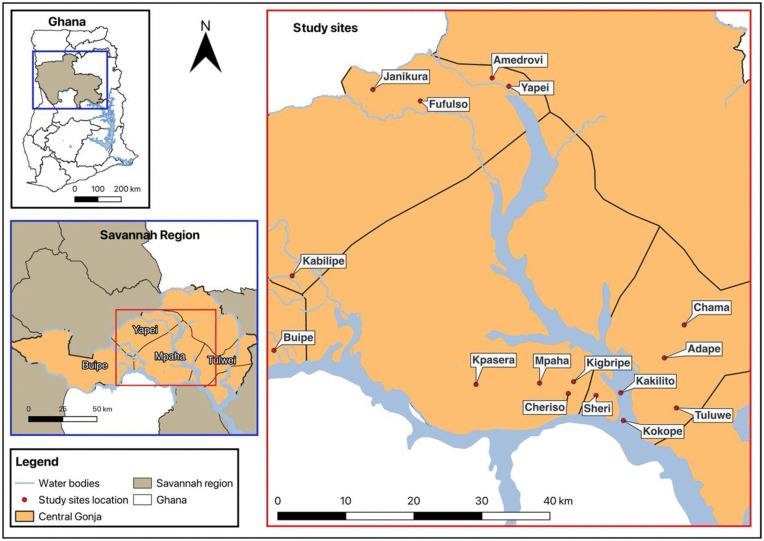
Map showing the Central Gonja District in the Savannah Region of Ghana, indicating the major communities and health facilities where sampling was conducted with base map adopted from the Regional Health Directorate Health Information files, Ghana Health Service.

This study was conducted in the central Gonja district of the Savannah region in Ghana, formally the northern region, as shown in Fig 1. The district lies between longitudes 1˚5’W and 2˚58’W and latitudes 8˚32’N and 10˚2’N. The 2021 national population census reports a total of 116,873 inhabitants residing in the district (source: GSS 2021 PHC). The district has two major rivers, that is the Black and the White Volta, which coalesce/confluence at Kikale. The study area was purposively chosen because of the freshwater source, which is a favourable breeding ground for the intermediate host snail of schistosomes. The inhabitants of this district are mostly farmers, with majority of them either living closer to the rivers or are fishermen and the women are dominantly fishmongers. The district is mostly rural, with the capital, Buipe, being peri-urban. The central Gonja district was selected for this study because, despite being classified by the Country Health Information Platform (CHIP) of the WHO Expanded Special Project for Elimination of Neglected Tropical Diseases (ESPEN) as having a moderate schistosomiasis prevalence (10–49%) [28] epidemiological data from northern part of Ghana remain limited. The study therefore sought to generate evidence from this underrepresented region and contribute to addressing existing knowledge gaps regarding schistosomiasis transmission and disease burden.

### Study design and sampling

The study employed a quantitative approach using a cross-sectional design. A multi-stage sampling strategy was implemented. In the first stage, purposive sampling was used to select health facilities located near the two major rivers, the Black and the White Volta, as well as all referral polyclinics and the district hospital in the Central Gonja District. Initially, 24 health facilities were targeted out of 47 health facilities in the district, but only 19 consented to participate in the study. In the second stage, a stratified sampling technique was applied. Each participating facility was stratified into six professional categories based on staff roles: nursing, midwifery, diagnostics, prescribers, pharmacy, and public health. At the third stage, a simple random sampling method was used to select eligible healthcare workers from each stratum. Eligibility was based on having worked in the respective unit for at least six months. No additional exclusion criteria were applied beyond the eligibility requirement of a minimum of six months of service in the respective facility. All selected participants were enrolled in the study after providing informed consent. A self-administered questionnaire was used to collect data. The data collection was done between June 2024 and October 2024. The questionnaire (S1 Data) was subdivided into seven sections: Demographic Data, Facility Data, Knowledge data (knowledge indicators or familiarity with the signs and symptoms, modes of transmission, control methods and risk factors), Capacity of Health Facilities in Treating FGS, Laboratory Capacity to Diagnose schistosomiasis & FGS, Clinical Care of Health Workers on schistosomiasis and FGS.

### Sample size estimation

The study population comprised health workers from selected health facilities in the district who are in close proximity to the volta rivers and are involved in clinical care, diagnosis, or management of reproductive and infectious diseases such as schistosomiasis. Based on facility staff lists and administrative records, an estimated total of 354 eligible health workers were identified across the selected facilities and invited to participate in the study. This number therefore represents the target population of eligible respondents. Sample size was estimated using Slovin’s formula (Slovin, 1960), which is commonly applied in survey-based studies when the population size is known and limited information is available on population variability. The formula provides an estimate of the minimum sample size required to achieve a specified margin of error:


$$\begin{array}{c} \text{n }=\;\frac{N}{\left(\text{1}\;+\text{ N}e^{\text{2}}\right)} \end{array}$$

Where n = Sample size

N= Population size = 354

e = Acceptable margin of error = 0.04 (4%)

Using this formula, a minimum sample size of 226 respondents was calculated. At the end of data collection however, 237 completed questionnaires were received, exceeding the minimum required sample size and representing a response rate of approximately 67% of the eligible population.

### Data collection

In each unit within the health facilities, all eligible staff members were approached and invited to participate. Recruitment involved visiting multiple units within facilities and obtaining informed consent from available and eligible individuals. All consenting staff who met the inclusion criteria were enrolled consecutively until the end of the data collection period. An open-access data collection tool, Kobo Collect [29] was shared with all the staff in each unit using a generated online form for them to respond to the questionnaire. The questionnaire was subjected to content validity assessment by a panel of five subject matter experts in public health and clinical practice, who reviewed the items for clarity, relevance and representativeness. Following expert review, the questionnaire was piloted among 23 healthcare workers at St. Anne Hospital, Damongo, who were not included in the main study. Feedback from both the expert review and the pilot testing informed minor wording revisions to improve clarity before the main data collection commenced.

### Data analysis

The collected data in Kobo Collect was downloaded as an Excel file, cleaned and uploaded into Statistical Package for Social Sciences (SPSS) version 20 for analysis. Assessment of schistosomiasis knowledge was based on responses to a series of structured knowledge questions addressing disease transmission, symptoms, prevention and treatment. Each correct response was awarded one point, while incorrect or “don’t know” responses were assigned zero points. Individual knowledge scores were obtained by summing the total number of correct responses. The scores were converted into percentages of the maximum obtainable score for schistosomiasis [27] and FGS [24]. Participants who scored 50% or higher were categorised as having good knowledge, whereas those who scored below 50% were categorised as having poor knowledge as used by Naabil et al., [30]. This categorisation was used for subsequent descriptive and inferential statistical analyses. Summarised descriptive statistics (frequency values and percentages) and crosstabulations were obtained for the Knowledge variables and the respondents’ demographic profile. Contingency tables were created, and Pearson’s chi-square test (χ2) was used to analyse nominal variables and to examine the association of selected variables with the two main outcome variables of interest; knowledge of schistosomiasis and knowledge of female genital schistosomiasis and considered statistically significant at a P < 0.05.

## Results

### Demographic characteristics

A total of 237 healthcare workers participated in the study, with a mean age of 31.6 ± 4.2 years (median = 31). Slightly more males than females were enrolled. Most respondents held certificate or diploma qualifications, and nurses constituted the largest professional group, followed by public health staff and midwives. The majority of participants were early in their careers, with nearly two-thirds having ≤3 years of professional experience. Respondents were drawn from 19 out of 24 eligible health facilities across the district, predominantly from the district hospital and polyclinics. All facilities were primary-level facilities. Detailed characteristics are presented in Table 1.

**Table 1:** Socio-demographic Characteristics of Health Workers in the Central Gonja District.

Characteristics	Description	Frequency	Percent (%)
Age	Mean ± Std. Deviation = 31.6 ± 4.178	Median = 31	Mode = 29
Gender	Female	113	47.7
	Male	124	52.3
Educational Level	Certificate	107	45.1
	Bachelor’s Degree	44	18.6
	Diploma	84	35.4
	Master’s Degree	2	0.8
Job Title Grouping	Diagnostic Staff	16	6.8
	Midwives	24	10.1
	Nurses	134	56.5
	Pharmacy Staff	10	4.2
	Prescribers	10	4.2
	Public Health Staff	43	18.1
Tenure Categories	Entry Level (0–3 yrs)	154	65
	Early Career Level (4–8 yrs)	58	24.5
	Mid-Career Level (9–13 yrs)	21	8.9
	Experienced Career Level(14–18yr)	3	1.3
	Highly Experienced Career Level (≥19 yrs)	1	0.4
Facility	Adape CHPS	3	1.3
	Amedrovi CHPS	1	0.4
	Bonyase CHPS	2	0.8
	Buipe Polyclinic	62	26.2
	Central Gonja District Hospital (CGDH)	88	37.1
	Chama CHPS	3	1.3
	Cheriso CHPS	1	0.4
	Fufulso Health Center	14	5.9
	Holistic Medicare	1	0.4
	Janikura CHPS	2	0.8
	Kigbripe CHPS	1	0.4
	Kokope CHPS	2	0.8
	Kpasera CHPS	3	1.3
	Mpaha Health Centre	25	10.5
	Sheri CHPS	2	0.8
	Tuluwe CHPS	3	1.3
	Yapei Polyclinic	18	7.6
	Yapei Quarters CHPS	4	1.7
	Yapei Yipala CHPS	2	0.8
**Respondents from Facility Type**	CHPS	29	11.2
	District Hospital	88	37.1
	Health Centre	39	16.5
	Polyclinic	80	33.8
	Private Health Facility	1	0.4
**Facility Type (N = 19)**	CHPS	13	68.4
	Polyclinic	2	10.5
	District Hospital	1	5.3
	Private Health Facility	1	5.3
	Health Centre	2	10.5

### Awareness and knowledge of schistosomiasis and FGS

**Fig 2:**
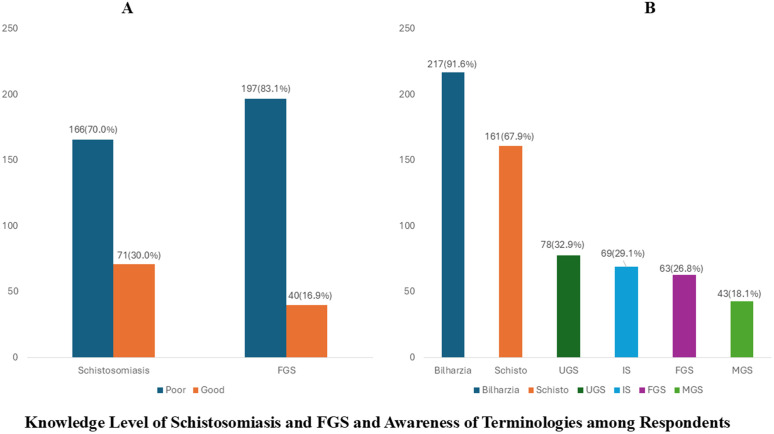
A: Knowledge level of schistosomiasis and female genital schistosomiasis; B: Awareness of schistosomiasis-related terminologies among respondents in the Central Gonja District, Ghana. *FGS - Female Genital Schistosomiasis, Schisto – Schistosomiasis, UGS - Urogenital Schistosomiasis, IS – Intestinal Schistosomiasis, MGS – Male Genital Schistosomiasis.

Fig 2 highlights the generally low knowledge levels of schistosomiasis and FGS among health workers in the Central Gonja District. Of the 237 respondents, 30.0% (n = 71) demonstrated good knowledge of schistosomiasis, while the majority, 70.0% (n = 166), exhibited poor knowledge. Similarly, knowledge of FGS was limited, with 16.9% (n = 40) showing good understanding, whereas 83.1% (197) had poor knowledge. The figure also shows the number of respondents who knew and had heard of the various forms of schistosomiasis. As it is commonly known, Bilharzia was selected by 91.6% of the respondents while 67.9% selected knowing schistosomiasis. We enquired if respondents knew schistosomiasis to be the same as bilharzia, and 70.5% affirmed. About 32.9% and 29.1% knew of Urogenital Schistosomiasis and Intestinal Schistosomiasis, respectively. Only 26.6% and 18.1% knew about FGS and MGS, respectively.

For enhancing awareness of FGS among health workers, respondents suggested multiple strategies. Popular approaches were to conduct “workshops” and “in-service training” sessions to “educate and enlighten staff” on FGS, its effects and management. Additionally, respondents emphasised “health education” through “public education,” such as community “durbars” and media engagement, along with resources like “fliers”, “clinical presentations” and “posters” to reinforce knowledge and visibility. They also suggested “curriculum adjustments” in training institutions and interprofessional meetings as essential measures to foster a comprehensive understanding and proactive engagement with FGS in healthcare settings.

### Awareness of the Association Between FGS, HIV and HPV Risk by Job Category

Table 2 highlights notable variations in awareness of FGS as a risk factor for HIV and HPV infection across different health worker cadres. Overall, awareness of the association between FGS and HIV appears higher than that for HPV, suggesting uneven understanding of the broader sexual and reproductive health implications of the disease. Clinical cadres with more direct patient contact, particularly nurses and midwives, demonstrated relatively better awareness compared to other groups, while knowledge gaps were evident among non-clinical and prescribing staff. The substantial proportion of non-responses across cadres further suggests uncertainty or limited exposure to information on FGS-related comorbidities.

**Table 2:** Awareness of the Association Between FGS, HIV and HPV Risk by Job Category.

Job Title Category	Are you aware FGS can increases the risk of contracting HIV?			Are you aware that FGS may increase the risk of HPV infection?			Total
	Yes	No	No Response	Yes	No	No Response	
Diagnostic Staff	0	0	16	0	0	16	16
Midwives	7	1	16	3	5	16	24
Nurses	37	9	88	31	15	88	134
Pharmacy Staff	0	2	8	0	2	8	10
Prescribers	2	2	6	0	4	6	10
Public Health Staff	5	3	35	3	5	35	43
Total	51	17	169	37	31	169	237

### Knowledge level of healthcare workers on schistosomiasis and female genital schistosomiasis

Overall knowledge of schistosomiasis and FGS among healthcare workers was low, with slightly higher scores for schistosomiasis and marked variability across respondents. As shown in Table 3, knowledge levels varied across demographic and professional characteristics; however, gender, educational level, and job title were not significantly associated with knowledge of either condition. In contrast, professional experience was significantly associated with FGS knowledge, with lower knowledge observed among entry-level healthcare workers and higher levels among more experienced staff. No significant association was observed between tenure and schistosomiasis knowledge.

**Table 3:** Association of Schistosomiasis and FGS Knowledge and Demographic Characteristics.

Description	Characteristics	Total	%	Schistosomiasis Knowledge Level (Mean Score ± SD = 10.02 ± 7.315)						Female Genital Schistosomiasis Knowledge Level (Mean Score ± SD = 4.02 ± 6.509)					
				Good	%	Poor	%	X^2^	P-value	Good	%	Poor	%	X^2^	P-value
Gender								1.196	0.274					1.13	0.286
	Female	113	47.7	30	26.5	83	73.5			16	14.2	97	85.8		
	Male	124	52.3	41	33.1	83	66.9			24	19.4	100	80.6		
Educational Level								1.258	0.739					1.95	0.583
	Certificate	107	45.1	29	27.1	78	72.9			18	16.8	89	83.2		
	Bachelor’s Degree	44	18.6	13	29.5	31	70.5			6	13.6	38	86.4		
	Diploma	84	35.4	28	33.3	56	66.7			15	17.9	69	82.1		
	Master’s Degree	2	0.8	1	50.0	1	50.0			1	50.0	1	50.0		
Job Title Grouping								10.034	0.074					5.4	0.369
	Diagnostic Staff	16	6.8	4	25.0	12	75.0			5	31.3	11	68.8		
	Midwives	24	10.1	4	16.7	20	83.3			2	8.3	22	91.7		
	Nurses	134	56.5	39	29.1	95	70.9			21	15.7	113	84.3		
	Pharmacy Staff	10	4.2	7	70.0	3	30.0			1	10.0	9	90.0		
	Prescribers	10	4.2	3	30.0	7	70.0			3	30.0	7	70.0		
	Public Health Staff	43	18.1	14	32.6	29	67.4			8	18.6	35	81.4		
Tenure Categories								8.42	0.077					15.6	0.003
	Entry Level (0–3 yrs)	154	65.0	43	27.9	111	72.1			23	14.9	131	85.1		
	Early Career Level (4–8 yrs)	58	24.5	17	29.3	41	70.7			11	19.0	47	81.0		
	Mid-Career Level (9–13 yrs)	21	8.9	8	38.1	13	61.9			3	14.3	18	85.7		
	Experienced Career Level(14–18yr)	3	1.3	3	100.0	0	0.0			3	100.0	0	0.0		
	Highly Experienced Career Level (≥19 yrs)	1	0.4	0	0.0	1	100.0			0	0.0	1	100.0		

### Facility’s Capacity for Diagnosing and Treating FGS

Significant disparities existed in laboratory and treatment capacity across healthcare facilities as shown in Table 4. Laboratory services were largely limited to higher-level facilities, while most CHPS compounds and several health centres lacked on-site laboratories. Where laboratories were available, diagnostic capacity was restricted to basic microscopy, with no facility equipped for definitive diagnosis of FGS. Availability of praziquantel was low and only sparingly available in higher-level facilities, indicating limited treatment readiness across the district. Notably, no facility had verified access to a colposcope, highlighting a critical gap in infrastructure for FGS diagnosis.

**Table 4:** Availability of Laboratories and Schistosomiasis Medication by Facility.

Facility Name	Does your facility have a laboratory?			Do you readily have medication for treating schistosomiasis in your facility?			Total
	No Response	No	Yes	No Response	No	Yes	
Adape CHPS	0	3	0	1	2	0	**3**
Amedrovi CHPS	0	1	0	1	0	0	**1**
Bonyase CHPS	0	2	0	1	1	0	**2**
Buipe Polyclinic	0	0	62	23	38	1	**62**
CGDH	1	0	87	38	41	9	**88**
Chama CHPS	0	3	0	1	2	0	**3**
Cheriso CHPS	0	1	0	1	0	0	**1**
Fufulso Health Centre	0	14	0	7	6	1	**14**
Holistic Medicare	0	0	1	1	0	0	**1**
Janikura CHPS	0	2	0	2	0	0	**2**
Kigbripe CHPS	0	1	0	0	1	0	**1**
Kokope CHPS	0	2	0	1	1	0	**2**
Kpasera CHPS	0	3	0	2	1	0	**3**
Mpaha Health Centre	1	0	24	12	2	11	**25**
Sheri CHPS	0	2	0	0	2	0	**2**
Tuluwe CHPS	0	3	0	2	1	0	**3**
Yapei Health Centre	0	0	18	11	7	0	**18**
Yapei Quarters CHPS	0	4	0	1	3	0	**4**
Yapei Yipala CHPS	0	2	0	0	2	0	**2**
**Total**	**2**	**43**	**192**	**105**	**108**	**22**	**237**

### Association between job categories and diagnostic capacities, knowledge and skills for identifying and managing Female Genital Schistosomiasis

Table 5 summarises responses from clinical staff involved in direct patient care regarding FGS management and diagnostic practices. While praziquantel was the most frequently identified treatment, a substantial proportion of respondents reported incorrect treatments or were unsure how FGS should be managed. Consideration of FGS in women presenting with symptoms suggestive of STIs was uncommon, and most respondents reported difficulty differentiating FGS from STIs and other vaginal infections. Similarly, FGS was infrequently included in differential diagnoses for vaginal infections with STI-like presentations. Nurses and midwives constituted the majority of respondents and accounted for most of the responses reflecting limited knowledge and awareness, highlighting critical gaps in frontline capacity for FGS recognition and management.

**Table 5:** Treatment and Consideration of Female Genital Schistosomiasis by Job Title/Occupation.

Job Title/Occupation	How do you treat FGS?					Do you consider FGS when female clients present with symptoms of STIs?			Are you able to differentiate FGS from STIs and other vaginal infections?			Do you consider FGS during differential diagnosis when clients present with vaginal infections similar to STIs?			Total
	No Response	PZQ	CTX	FLZ	Don’t know	No Response	No	Yes	No Response	No	Yes	No Response	No	Yes	
Facility In-Charge	0	1	0	1	1	0	0	3	0	3	0	0	0	3	3
Medical Doctor	0	2	0	0	0	0	0	2	0	1	1	0	0	2	2
Midwife	3	9	2	2	8	3	17	4	3	17	4	3	16	5	24
Nurse	18	22	6	5	8	18	21	20	18	24	17	18	23	18	59
Nurse Practitioner	1	3	0	0	0	1	1	2	1	0	3	1	2	1	4
Physician Assistant	0	4	0	0	0	0	3	1	0	4	0	0	4	0	4
Total	22	41	8	8	17	22	42	32	22	49	25	22	45	29	96

* PZQ = Praziquantel (Anthelmintic) CTX = Ceftriaxone (Antibiotic) FLZ = Fluconazole (Antifungal).

### Perceptions of Respondents on Genital Infections and Schistosomiasis

As shown in Table 6, attitudes toward clients presenting with genital infections varied across professional groups, with a minority of respondents expressing stigmatising perceptions. Such views were more frequently reported among frontline clinical cadres, particularly nurses and midwives, while they were uncommon among prescribers and diagnostic staff. In contrast, there was near-universal agreement among respondents that schistosomiasis constitutes a public health concern and that FGS poses a threat to women’s reproductive health. Similarly, overwhelming consensus was observed regarding the need for increased awareness creation and education on FGS across all professional groups.

**Table 6:** Perceptions of Respondents on Genital Infections and Schistosomiasis.

Job Title Grouping	Do you consider clients to be promiscuous when they present with genital infections?			Do you consider Schistosomiasis to be a public health concern?			Do you consider FGS to be a threat to women’s reproductive health?			Do you see the need for awareness creation and education on FGS?			Total
	Did Not Respond	No	Yes	Did Not Respond	No	Yes	Did Not Respond	No	Yes	Did Not Respond	No	Yes	
Public Health Staff	37	3	3	1	0	42	1	1	41	1	0	42	43
Prescribers	3	7	0	0	1	9	0	0	10	0	0	10	10
Diagnostic Staff	3	8	5	0	1	15	0	0	16	0	0	16	16
Midwives	0	18	6	0	1	23	0	1	23	0	1	23	24
Nurses	67	46	21	0	20	114	0	3	131	0	4	130	134
Pharmacy Staff	10	0	0	0	1	9	0	0	10	0	0	10	10
**Total**	**120**	**82**	**35**	**1**	**24**	**212**	**1**	**5**	**231**	**1**	**5**	**231**	**237**

## Discussion

This study assessed the knowledge level of health workers in the central Gonja district on FGS following an ongoing prevalence study. FGS manifestations are often misdiagnosed as sexually transmitted infections or other gynaecological conditions due to overlapping clinical features and limited awareness among healthcare professionals. The clinical and socioeconomic impacts of FGS are profound. Despite these implications, the knowledge and recognition of FGS among healthcare professionals remain insufficient, contributing to misdiagnoses.

Health workers had poor knowledge of schistosomiasis and FGS. However, the mean score for schistosomiasis was better than FGS. Bilharzia was the most common name referred to by respondents as schistosomiasis with a lesser percentage of them not knowing or unsure. More respondents knew about urinary schistosomiasis as compared to FGS and MGS which aligns with other similar studies from Ghana, Tanzania and the DRC [1,27,31–34]. This was posited to be the case by Wambui et al. [1] because blood in urine is the hallmark symptom for identifying urinary schistosomiasis. In this study, it was the most highlighted symptom of schistosomiasis. Awareness level of urinary, intestinal, female genital and male genital schistosomiasis were respectively low in this study. This reflects the general trend where more people are aware of urinary schistosomiasis than FGS [1,24,31] due to a lot of awareness creation and community efforts towards eliminating urinary schistosomiasis compared to FGS which, until recently, was not talked about much [35]. Relatively low FGS awareness among health workers has been reported in studies in Ghana and Tanzania [24,32].

This study also assessed the association between FGS and schistosomiasis knowledge levels and various demographic factors, including job title, educational level, length of service, and gender. Only length of service showed a statistically significant association with FGS knowledge level (p = 0.003). This suggests that healthcare workers with longer service durations may have encountered FGS cases more frequently, even if they had previously overlooked them. Some gynaecological clinical staff recalled instances of women from riparian communities presenting with persistent vaginal infections that did not resolve with standard treatment, a realisation that emerged after the questionnaire was administered and informal education on FGS was provided. Poor awareness of FGS could contribute to misdiagnoses, affecting women’s reproductive health, increasing financial and mental health burdens, and ultimately hindering progress toward the WHO’s 2030 schistosomiasis elimination goal.

FGS has been associated with increased susceptibility to HIV [15, 36] and HPV [37] infection among women in schistosomiasis-endemic regions. Knowledge of this would help health workers in considering FGS when women present with genital infections. Proper diagnosis and treatment would help resolve FGS associated genital lesions and reduce the risk of contracting HIV. We therefore collated data on the awareness of FGS being a cofactor in increasing the risk of HIV and HPV infection. The results showed that majority of respondents were aware of the association between FGS and HIV acquisition as compared to FGS and HPV infection. This disparity in awareness may stem from the greater emphasis in public health campaigns and literature on the link between HIV and other health conditions, including FGS, compared to HPV. HIV’s widespread attention as a global health issue likely contributes to higher recognition of its associations among healthcare workers as compared to HPV. Healthcare workers often mistake the symptoms and clinical signs of FGS for those of STIs due to their similarities [38] however, FGS is rarely included in their differential diagnoses or treatment plans unless the patient fails to respond to the initial therapy. Likewise, in a study conducted by Kukula et al. [24] in Ghana, adolescent girls exhibiting symptoms of FGS were often referred for treatment for STIs. This also presents another avenue for education to help reduce the risk of transmission in these areas.

After acquiring knowledge on FGS, health workers need resources to help diagnose and successfully treat. Colposcopy is one of the diagnostic methods for the diagnosis of FGS. It helps to visualise lesions in the vagina and then compare with the WHO FGS atlas as a guide [39]. All facilities that took part in this study did not have a colposcope. This finding was the case in Wambui et al and Mazigo et al’s study [1,32] and other studies in sub-Saharan Africa [2,17]. The lack of such an important diagnostic tool impedes diagnosis even if FGS is considered in differential diagnosis. The reason for not having a colposcope by these facilities could be attributed to its high cost. An alternative diagnostic method will be PCR of cervicovaginal samples in the laboratory, but only tertiary and some secondary health facilities have such sophisticated equipment which is also expensive. Availability of praziquantel was also rare in the various facilities just like in studies conducted by Christinet et al., [2] Engels et al., [17] and Mazigo et al., [32]. The head of one of the health facilities intimated that two pregnant women diagnosed with urinary schistosomiasis were once left to their fate due to lack of medication even at the district hospital.

Staff who directly managed clients answered questions on how FGS is treated. Most of them chose praziquantel, the drug of choice, but others did not know, and some also chose antibiotics and antifungals, which are for other vaginal infections. This indicates that mismanagement, whether due to a lack of knowledge about the appropriate treatment or the prescription of antibiotics or antifungals for misdiagnosed FGS cases, could place a significant burden on both patients and the healthcare system. Additional questions assessed whether health workers differentiated FGS from STIs and other vaginal infections. Most respondents said “No,” and some abstained, indicating they did not. Only a few considered FGS when diagnosing genital symptoms. This implies that a lot of such cases will be missed and/or misdiagnosed, thereby causing future complications for these women. Regarding education on FGS, 92.8% of respondents have never had any form of education on the subject matter as was the case in a study conducted by Azanu et al., [27] in Ghana. FGS is a relatively recent focus of attention, even among clinicians, and is not yet included in Ghana’s educational institutions’ curriculum. During the FGS Accelerated Scale Together (FAST) package study, an in-person Continuous Professional Development programme was organised for subject matter experts from the Ghana College of Physicians and Surgeons, nursing training colleges, and selected university faculties, with the aim of introducing FGS into their curricula [40]. As a result, healthcare workers can only learn about it through specialised workshops and research articles. In contrast, schistosomiasis is covered in the curricula of most senior high schools and healthcare training institutions [27] in Ghana.

The perception of healthcare workers regarding FGS and schistosomiasis reveals notable trends in understanding and attitudes across various job roles. While most healthcare staff did not overtly associate genital infections with promiscuity, some degree of stigma was still evident, highlighting the need for training in non-judgmental and compassionate care. Similar patterns have been reported in STI studies in Ghana by Sawyer and Seidu et al., [41,42] where adolescent girls often experience shame and stigma from both communities and healthcare providers when seeking care for genital symptoms [43]. Given that FGS frequently presents with symptoms that mimic STIs, affected girls and women may face comparable stigmatisation, potentially delaying care-seeking and appropriate diagnosis. Furthermore, a strong majority of healthcare workers acknowledge both schistosomiasis as a public health concern and FGS as a significant threat to women’s reproductive health. This shared awareness likely reflects the widespread recognition of schistosomiasis’s impact on public health and emphasises the need for targeted interventions. Importantly, respondents also see the need for awareness creation and education on FGS, suggesting a high level of commitment within the healthcare community to support advocacy and outreach efforts. Together, these insights point toward a robust foundation for enhancing FGS and schistosomiasis education and implementing stigma-reducing strategies within clinical practice.

These indications suggest that future research should extend beyond healthcare workers to explore patients’ perceptions, beliefs, and care-seeking behaviours related to FGS. Understanding how women interpret symptoms, experience stigma and navigate health services will be critical for improving early recognition, appropriate referral and effective community-based interventions. Integrating patient perspectives with provider-focused assessments will provide a more comprehensive understanding of barriers to FGS diagnosis and management.

### Limitations of the study

Self-administered electronic questionnaire: The study relied on a self-administered electronic questionnaire, which may have introduced response bias, including misunderstanding of questions, socially desirable responses, or incomplete answers. In addition, variations in digital literacy, possession of a smart device and internet access across facilities could have affected participation and the quality of responses. To mitigate this, the tool was pre-tested, refined for clarity and deployed with clear instructions to minimise misunderstanding.

Unequal staff distribution across facilities: Health facilities in the district had widely varying staff sizes, ranging from as few as 1–3 staff in some facilities to several dozens and even hundreds in others. This imbalance may have led to overrepresentation of responses from larger facilities and underrepresentation from smaller ones, potentially affecting the generalisability of the findings to all facilities in the district. To address this, the questionnaire link was disseminated widely to both large and small facilities and responses were reviewed by facility size during analysis.

Voluntary participation and non-response bias: Participation was voluntary and some eligible health workers chose not to respond or abstained from answering certain questions. This may have introduced non-response bias, as those with greater interest or awareness of FGS may have been more likely to participate, potentially influencing estimates of knowledge and diagnostic competence. To reduce this, multiple reminders were sent, facility heads were engaged to encourage participation and missing or abstained responses were transparently reported and considered during interpretation.

Despite these limitations, the study provides important insights into health workers’ knowledge, awareness, and diagnostic considerations regarding FGS in the district. The study offers valuable baseline information that can inform targeted training, awareness creation, and improvements in the recognition and management of FGS among health workers, while also highlighting areas for future research using more comprehensive sampling and data collection approaches.

## Conclusion

The study revealed a significant knowledge gap on schistosomiasis and female genital schistosomiasis among healthcare professionals in the Central Gonja District. This raises concerns about potential missed or misdiagnosed cases being classified as sexually transmitted infections. Addressing this gap is crucial, as it highlights the need for targeted capacity-building initiatives, such as workshops, in-service training programs, and clinical conferences, particularly for frontline healthcare staff involved in patient care. To enhance awareness and accurate diagnosis of FGS, it is recommended that future research focuses on intervention-based training programs and subsequent knowledge assessments. Such studies could also investigate the prevalence of FGS among genital infections reported at various health facilities, providing valuable data to guide public health interventions and improve patient outcomes. Although the findings provide valuable baseline evidence, they should be interpreted with caution due to the use of a self-administered electronic questionnaire, unequal staff distribution across facilities and the potential for non-response bias associated with voluntary participation. Nevertheless, the study offers important insights into healthcare workers’ knowledge and diagnostic capacity regarding FGS in this underrepresented district and can inform future training and research initiatives.

## Supporting information

S1 Data Questionnaire. The questionnaires used for data collection.
(DOCX)

S2 Data Research Data. As requested for data transparency, this is the data collected.
(XLSX)
