# Supplementary material for: Diagnostic competence and health worker knowledge of female genital schistosomiasis management in a rural Ghanaian district
Source: PLoS Negl Trop Dis. 2026 Jun 30;20(6):e0013638. doi: 10.1371/journal.pntd.0013638 (PMC13340770; doi:10.1371/journal.pntd.0013638)
Supplement: S1 Data — The questionnaires used for data collection. (DOCX) [file pntd.0013638.s001.docx]

**QUESTIONNAIRE**

**UNIVERSITY FOR DEVELOPMENT STUDIES**

**SCHOOL OF MEDICINE AND HEALTH SCIENCES**

**DEPARTMENT OF CLINICAL MICROBIOLOGY**

**RESEARCH TOPIC: EPIDEMIOLOGY AND CHARACTERIZATION OF SCHISTOSOMIASIS IN THE CENTRAL GONJA DISTRICT OF GHANA**

**Health Worker Knowledge Assessment on Female Genital Schistosomiasis**

**DEMOGRAPHIC DATA**

1. Age:____________
2. Gender: Male ( ) Female( )
3. Education level: Certificate( ) diploma( ) degree( ) Masters( ) PhD( )
4. Length of Service:___________
5. **Job Title/ Occupation**: Facility In Charge( )Physician Assistant( ) Pharmacist ( ) Pharmacy Technician ( ) Dispensing Assistant ( ) Medical Doctor ( ) Nurse Practitioner( ) Nurse ( ) Enrolled Nurse ( ) Medical Laboratory Scientist ( ) Medical Laboratory Technician( ) Medical Laboratory Assistant ( ) Midwife ( ) Radiographer ( ) Sonographer ( ) X-ray technician ( ) Traditional Medicine Practitioner ( ) Disease Control Officer ( ) Health Promotion Officer ( ) Health Educator ( ) Public Health Officer ( ) Public Health Nurse( ) Community Health Officer ( ) Health Information Officer ( ) Field Technician ( ) Community Health Nurse ( ) Health Research Officer( )

**FACILITY DATA**

1. Name of Facility: Central Gonja District Hospital ( ) Buipe Polyclinic ( ) Holistic Medicare ( ) Buipe CHPS ( ) Mpaha Health Centre ( ) Kpasera CHPS ( ) Gbrigi CHPS ( ) Kpabuso CHPS ( ) Kokope CHPS ( ) Kigbripe CHPS ( ) Sheri CHPS ( ) Cheriso CHPS ( ) Tuluwe CHPS ( ) Adape CHPS ( ) Digma CHPS ( ) Chama CHPS ( ) YAPEI HEALTH CENTRE ( ) Fufulso Health Center ( ) Yapei Quarters CHPS ( ) Amedrovi CHPS ( ) Janikura CHPS ( ) Yapei Yipala CHPS ( ) Bonyase CHPS ( )
2. Kind of Facility: CHPS( ) Clinic( ) Polyclinic( ) District Hospital( ) Private Health Facility( )

**KNOWLEDGE**

1. Which of the following have you heard of? Schistosomiasis ( ) Bilharzia ( ) Urogenital Schistosomiasis( ) Intestinal Schistosomiasis ( ) Female Genital Schistosomiasis ( ) Male Genital Schistosomiasis( ) None of the above( )
2. Where did you first hear of any of the above? a. School ( ) b. On the Job ( ) c. Media (TV, radio, newspapers)( ) d. Internet ( ) e. Family or friends ( ) f. Other (please specify) ……………………………………………….
3. Is Bilharzia the same as schistosomiasis? a. Yes ( ) b. No ( ) c. Don’t know ( )
4. What is schistosomiasis?.....................................................................................................................
5. How is it locally called (specify language and how it’s called)…………………………….
6. Is schistosomiasis a common disease in this community? Yes ( ) b. No c. Don’t know (Hint: do you get clients complaining often about blood in urine or stool?)
7. Approximately how many cases have you encountered this year?..........................
8. What is the causative organism of schistosomiasis? a. Parasite ( ) b. Fungus c. Bacteria( ) d. Virus e. Don’t know ( )
9. How is schistosomiasis transmitted? A) Through contact with contaminated water B) Through the ingestion of contaminated food C) Through contact with infected soil (D) Through mosquito bites (e)Spiritual
10. Select all the symptoms of schistosomiasis you know. Cough ( ) Blood in urine( ) Joint pain( ) Dysuria( ) Frequency of urination( ) Loss of appetite( ) Abdominal pain( ) Night sweats( ) blood in stool( ) Genital Discharge( ) Fatigue( ) Vision Problems( ) Anemia( ) Skin rash( ) Diarrhoea( ) Muscle ache( ) Jaundice ( ) Headache( )
11. Are you aware urinary schistosomiasis can affect the reproductive system? a. Yes ( ) b. No ( )
12. What is Female Genital Schistosomiasis? a. A sexually transmitted infection affecting the female genitalia ( ) b. A parasitic disease caused by blood flukes that affect the female reproductive system ( ) c. A bacteria infection that causes vaginosis ( ) d. A fungal infection that causes vaginal discharge and itching ( ) e. Don’t know ( )
13. Select all signs and symptoms of Female Genital Schistosomiasis that apply. a. Contact bleeding ( ) b. Lower abdominal pain ( ) c. genital itching or burning sensation ( ) d. vaginal discharge ( ) e. Lower abdominal pain ( ) f. Urinary incontinence ( ) g. Irregular menstruation ( ) h. Don’t know ()
14. Select all methods used to diagnose Female Genital Schistosomiasis that you know. a. Colposcopy b. Vaginal examination c. Punch biopsy from the vagina d. PCR of vaginal sample e. medical history f. Stool microscopy g. urine microscopy h. Don’t know
15. How do the local people in this community diagnose female genital schistosomiasis? Select all that apply. Through physical symptoms(postcoital bleeding) ( )By traditional healers and herbalist ( ) Using self-reported symptoms and local knowledge ( ) Consulting with community health workers ( ) Through the presence of blood in urine ( ) Don’t know
16. Select all complications of female genital schistosomiasis that you know a. Infertility b. Miscarriage c. Ectopic Pregnancy d. Increased susceptibility to HIV e. Social and Psychological Consequence due to stigmatisation f. Malnutrition g. Don’t Know
17. Are you aware that FGS increases the risk of contracting HIV/AIDS? a. Yes ( ) b. No ( )
18. Are you aware that FGS may increase the risk of HPV infection? a. Yes ( ) b. No ( )

**CAPACITY OF HEALTH FACILITIES IN TREATING FGS**

1. Does your facility have a colposcope for vaginal examination? a. Yes ( ) b. No ( ) Don’t know ( )
2. Do you readily have medication for treating schistosomiasis in your facility? Yes ( ) No ()
3. If yes name the drug…………………………………………………
4. Have you had training on female genital schistosomiasis before? a. Yes ( ) b. No ( )
5. Does your facility have a laboratory? a. Yes ( ) b. No ( )

**LAB CAPACITY TO DIAGNOSE SCH & FGS** *(Skip logic – only to be answered by lab staff )*

1. Is your laboratory equipped with basic equipment to diagnose schistosomiasis? (microscope and or RDT for Sch diagnosis) a. Yes ( ) b. No ( )
2. Is your laboratory equipped with advanced equipment to diagnose schistosomiasis and female genital schistosomiasis? (PCR, ELISA, RDT, Histopathology and Cytology) a. Yes ( ) b. No ( )

**CLINICAL CARE** *(Skip logic – only to be answered by clinical staff that directly assess clients)*

1. How do you treat FGS? a. Antehemintic (e.g. Praziquantel)( ) b. antibiotics (e.g. ceftriaxone) c. antifungal (e.g. fluconazole)
2. Do you consider FGS when female clients present with symptoms of STIs/STDs? a. Yes () b. No ()
3. Are you able to differentiate FGS from STIs/STDs and other vaginal infections? a. Yes () b. No ()
4. Do you consider FGS during differential diagnosis when clients present with vaginal infections similar to STIs? a. Yes ( ) b. No ( )

**PERCEPTION OF HEALTH WORKERS ON SCH & FGS**

1. Do you consider clients to be promiscuous when they present with genital infections? Yes ( ) b. No
2. Do you consider Schistosomiasis to be a public health concern? a. Yes () b. No() c Don’t Know( )
3. Do you consider FGS to be a threat to women’s reproductive health? a. Yes ( ) b. No ( ) c Don’t Know
4. Do you see the need for awareness creation and education on FGS? a. Yes ( ) b. No ( ) c Don’t Know
5. Suggest ways to create awareness regarding FGS among health workers………………………………………………………………………………………………………………………………………………………………………………………………………
